# Biophysical ambiguities prevent accurate genetic prediction

**DOI:** 10.1038/s41467-020-18694-0

**Published:** 2020-10-01

**Authors:** Xianghua Li, Ben Lehner

**Affiliations:** 1 Centre for Genomic Regulation (CRG), The Barcelona Institute of Science and Technology, Dr. Aiguader 88, Barcelona, 08003 Spain; 2 Universitat Pompeu Fabra (UPF), Barcelona, Spain; 3 ICREA, Pg. Luis Companys 23, Barcelona, 08010 Spain

**Keywords:** Mutation, Quantitative trait, Systems biology, Epistasis

## Abstract

A goal of biology is to predict how mutations combine to alter phenotypes, fitness and disease. It is often assumed that mutations combine additively or with interactions that can be predicted. Here, we show using simulations that, even for the simple example of the lambda phage transcription factor CI repressing a gene, this assumption is incorrect and that perfect measurements of the effects of mutations on a trait and mechanistic understanding can be insufficient to predict what happens when two mutations are combined. This apparent paradox arises because mutations can have different biophysical effects to cause the same change in a phenotype and the outcome in a double mutant depends upon what these hidden biophysical changes actually are. Pleiotropy and non-monotonic functions further confound prediction of how mutations interact. Accurate prediction of phenotypes and disease will sometimes not be possible unless these biophysical ambiguities can be resolved using additional measurements.

## Abstract (web summary)

In quantitative genetics, it is widely assumed that mutations combine additively or epistasis can be predicted with statistical or mechanistic models. Here, the authors use the phage lambda repressor model to show how biophysical ambiguity and non-monotonic functions confound phenotypic prediction.

## Introduction

A fundamental challenge across diverse fields of biology including human genetics, animal and plant breeding, and evolutionary theory is to predict how changes in genotypes result in changes in phenotypes and fitness. Accurate prediction of phenotypes from sequence entails two sub-challenges: predicting the mutations that individually affect a trait of interest and by how much, and predicting the joint effects when multiple mutations are combined in an individual. Progress is being made in both systematically identifying^1–3^ and predicting^4–6^ the mutations that impact traits of interest. Moreover, the extent to which mutations combine additively or with genetic (epistatic) interactions is being systematically quantified across diverse systems and phenotypes^7,8^.

However, a more fundamental question remains that is not addressed in any of these studies. Even if we have perfect measurements of the individual effects of a set of mutations on a trait and a very good mechanistic understanding of a system, can we always predict what happens when two mutations are combined?

In this study, we use a simple biophysical system to address this question. We show that, for diverse biological systems, the answer to this question will often be no. The fundamental reason for this is that different combinations of biophysical parameters can give rise to the same phenotypic value^9^.

The phage lambda repressor, CI, is one of the best-understood proteins in biology and a classic model for gene regulation, protein biophysics and systems biology^10–14^. CI regulates transcription from two divergent promoters with well-established dose–response curves: it represses transcription from the P_R_ promoter via a monotonic function but induces and then represses transcription from the P_RM_ promoter via a non-monotonic peaked function. The molecular mechanisms that underlie these regulatory responses are well-understood^10,15,16^ and thermodynamic models that incorporate them accurately predict the behaviour of the system^17–20^. Specifically, Ackers’ statistical thermodynamic model predicts the probabilities of the ON and OFF configuration states of the P_R_ and P_RM_ promoters as a function of the total repressor concentration^17^. To predict how mutations that affect the stability of CI combine to affect gene regulation, Ackers’ model can be combined with a thermodynamic model of protein folding^19^.

**Fig. 1:**
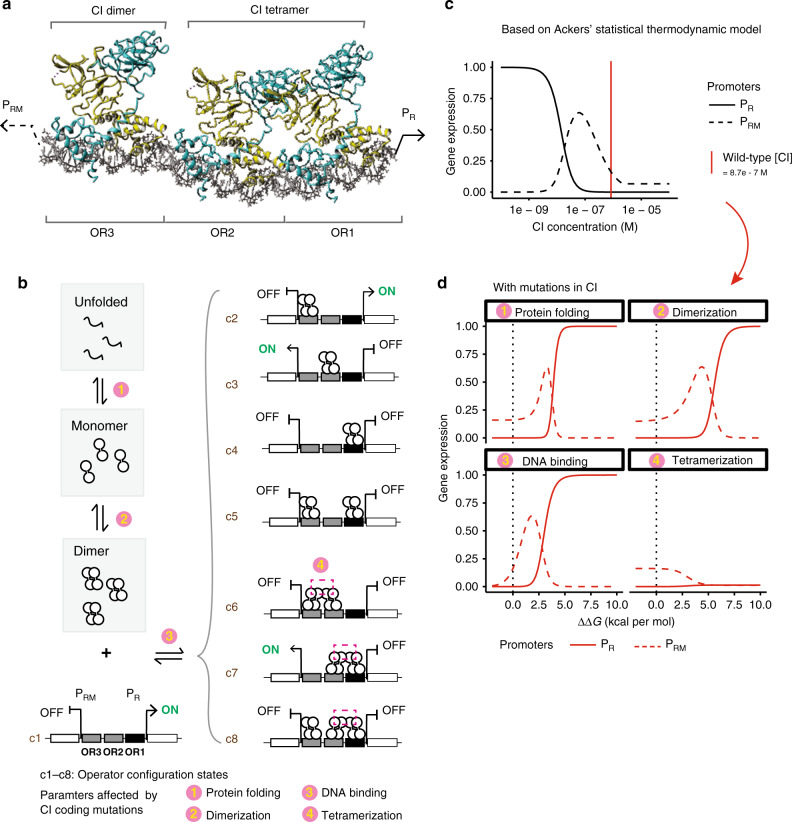
Genetic interactions in a transcription factor. **a** CI binds three operators as a dimer with two dimers also forming a tetrameric complex. Cyan and yellow distinguish the two monomers of each dimer. **b** Statistical thermodynamic model of gene regulation by the lambda repressor (CI). CI exists as unfolded, folded monomer, free dimer and dimers that are bound to operators. The partitioning of these molecules depends on Gibbs free-energy differences between states. **c** Dose–response curves of the P_R_ and P_RM_ promoters. **d** Mutations result in additive changes in the free energy of protein folding, dimerization, DNA binding and tetramerization. When only one free-energy term is altered, gene expression is altered by the eight plotted relationships. Dotted vertical black lines denote ∆∆*G* = 0 (wild type). See also Supplementary Fig. 1. Source data are provided as a Source data file.

Like most proteins^21^, CI is multifunctional: in order to regulate transcription it must fold correctly^22–25^, form a dimeric complex^26^, bind to DNA at multiple operator sites^27,28^ and also form a higher-order tetrameric complex^29,30^ on the genome (Fig. 1a). Mutations in CI can affect any of these biophysical activities, making CI a good model for investigating how mutations with different biophysical effects interact to alter cellular phenotypes.

However, mutations in a CI, like mutations in other proteins, can actually affect more than one biophysical parameter at the same time. For example, of 12 mutations that alter the binding affinity of CI to DNA, six (50%) also affected the stability of the protein^27,31–33^. Such biophysical pleiotropy is common, for example, mutations that alter enzymatic activity often reduce protein stability^34^. Similarly, mutations that alter protein binding affinities also frequently impact stability^31,35^ and in allosteric proteins changes in the affinity of binding at one site will alter the binding affinity at a second site^36^.

Here, using gene regulation by the lambda repressor model, we show that, even for a very simple biophysical system, it is often impossible to predict what happens when two mutations are combined even if we have perfect measurements of their effects on a trait. The cause of this apparent paradox is the one-to-many mapping between phenotypes and the underlying biophysical parameter changes that can cause them. When combining mutations, the outcome can be very different depending upon what these unidentified biophysical changes actually are. Our results illustrate how accurate genetic prediction of phenotypes and disease will often not be possible unless additional measurements are made to resolve the biophysical ambiguities in genotype–phenotype maps.

## Results

### Combining mutations in a thermodynamic model

To better understand how genetic variants with different biophysical effects combine to alter phenotypes, we investigated how mutations in a model transcription factor, the lambda repressor (CI), alter the expression of two target genes using an extensively validated thermodynamic model (Fig. 1b)^17–20^. We first considered mutations that affect the folding or stability of CI. Changes in protein stability are one of the most frequent effects of amino acid changes and a major cause of genetic disease^22–25^. The fraction of a protein in its natively folded state depends on the difference in Gibbs free energy (∆*G*) between its folded and unfolded states. Unless they are energetically coupled^37^, mutations have effects on stability that are additive at the level of free energy but non-additive for changes in protein concentration and expression from the P_R_ and P_RM_ promoters, which are our two phenotypic traits of interest (Fig. 1c, d)^19,38,39^.

### Genetic prediction for mutations affecting protein stability

**Fig. 2:**
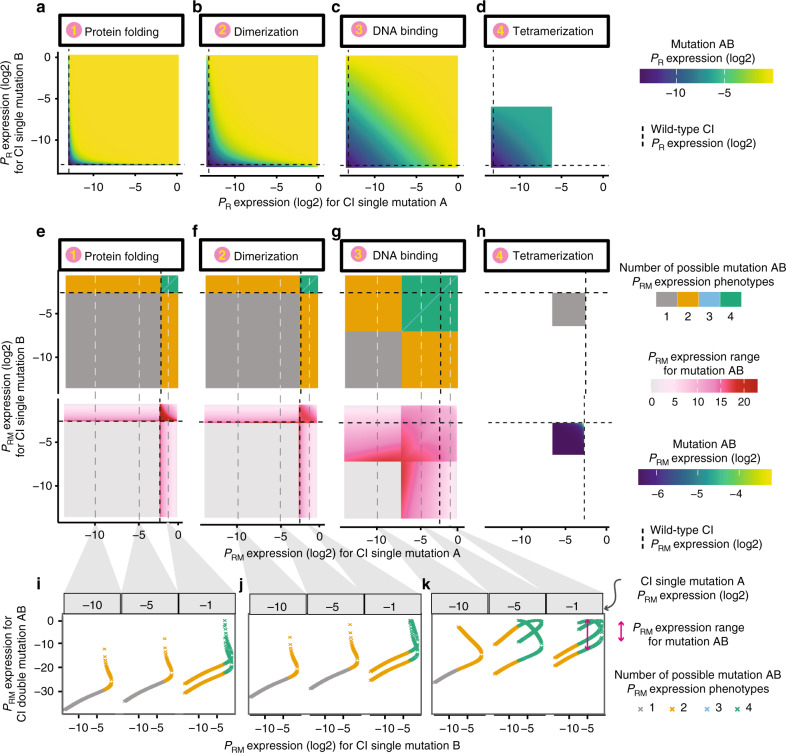
Non-monotonicity results in ambiguous phenotype prediction. **a**–**d** Double mutant P_R_ expression when combining CI mutations both affecting the same biophysical parameter: protein folding (**a**), dimerization (**b**), DNA binding (**c**) or tetramerization (**d**). **e**–**h** Double mutant P_RM_ expression when combining CI mutations affecting the same biophysical parameter: protein folding (**e**), dimerization (**f**), DNA binding (**g**) or tetramerization (**h**). Top row panels show number of possible P_RM_ expression phenotypes when combining two single mutant phenotypes. Bottom row panels (**e**–**g**) show the range of possible P_RM_ phenotypes. Bottom row of (**h**) shows P_RM_ expression since there is no ambiguous prediction. **i**–**k** Examples showing how three mutations with known P_RM_ expression phenotypes combine with second mutations with known phenotypes to result in up to four different expression levels in the double mutant. Source data are provided as a Source data file.

If two mutations that only affect protein stability are combined, the change in expression from P_R_ is often non-additive (i.e. there is substantial epistasis)^19^. However, the phenotype of the double mutant can normally be unambiguously predicted from the phenotypes of the two constituent single mutants because the free-energy-phenotype function is monotonic^40^ (Fig. 2a). The exception is when mutations have phenotypes that map to the top or bottom plateaus of the free-energy-phenotype function where the gradient approaches zero (Fig. 1d and Supplementary Fig. 1b–e) and measurement imprecision results in ambiguity in the underlying causal free-energy changes.

For expression from the P_RM_ promoter, however, this is not the case. Combining two mutations with measured effects on P_RM_ expression can result in more than one P_RM_ expression value, depending upon what the hidden underlying free-energy changes are^19,40^. The cause of this ambiguity in the phenotype of a double mutant is the non-monotonic input–output function of P_RM_ (Fig. 1c, d), which means that many phenotypic values can map to two different underlying changes in the free energy of protein folding (Fig. 1d). Thus, when combining mutations of known phenotypic effect, there can be up to four different valid phenotypic outcomes in the double mutant (Fig. 2e) and these outcomes can differ by almost the entire phenotypic range (Fig. 2e, i). Thus, even if mutations only affect protein folding, non-monotonic input–output functions and plateaus in free-energy-phenotype functions can make it impossible to predict how two mutations of known effect will combine to alter a phenotype.

### Mutations with other known biophysical effects

Mutations in proteins can, however, affect more than their stability. For example, mutations in CI can alter the binding affinity of the protein for itself (dimerization)^26^, its affinity for DNA^27,28^ and the affinity between two dimers to form a tetramer^29,30^. As for mutations affecting protein stability, mutations causing additive changes in the free energy of these molecular interactions (Fig. 1d) often combine to cause non-additive changes in expression from the two target promoters (Fig. 2b–d), generating substantial epistasis. However, for expression from P_R_ there is again no ambiguity in the double mutant phenotypes, with the exception of uncertainty created by imprecise measurements at the plateaus of the free-energy-phenotype functions (Fig. 1d and Supplementary Fig. 1b, c). However, as when combining mutations that only affect protein folding, pairs of mutations of known phenotypic effect that both only affect either dimerization or DNA binding can combine to have up to four different P_RM_ phenotypes as double mutants (Fig. 2f–k, Supplementary Fig. 2). Similar conclusions are obtained if the two mutations individually affect two different (but known) biophysical parameters: P_RM_ expression often cannot be unambiguously predicted, including when one of the mutations affects tetramerization (Supplementary Fig. 2b, c), while P_R_ expression can always be predictable without ambiguity (Supplementary Fig. 2a).

### Prediction for mutations with unknown biophysical effects

So far, we have considered cases where we know the identity of the biophysical parameter affected by each mutation. But normally we actually do not know which biophysical property of a protein is altered by a mutation. For example, any measured change in P_R_ expression resulting from a mutation in CI could be caused by a mutation that affects folding, DNA binding or dimerization (Fig. 1d, mutations that affect tetramerization have a more limited range of phenotypic outcomes).

**Fig. 3:**
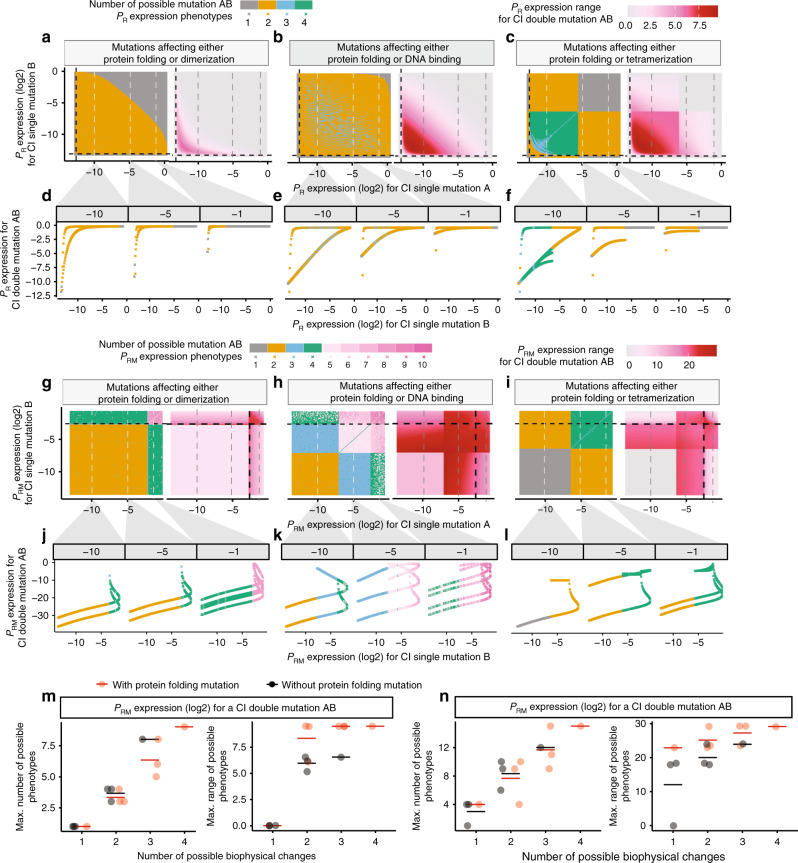
Biophysical ambiguity prevents phenotype prediction. **a**–**c** P_R_ expression when combining two mutations that affect either protein folding (Mutation A) or another biophysical parameter (Mutation B) but not both: dimerization (**a**), DNA binding (**b**) or tetramerization (**c**). Number (left) and range (right) of possible double mutant P_R_ phenotypes (left). **d**–**f** Examples showing how a mutation with a known phenotype combines with other mutations, leading to 1 to 4 possible double mutant P_R_ expression levels. **g**–**i** Number (left) and range (right) of double mutant P_RM_ expression levels when mutations can affect folding or another biophysical parameter. **j**–**l** Examples showing how a mutation with a known P_RM_ phenotype can combine with other mutations to result in many different P_RM_ phenotypes. **m**, **n** Maximum number (left) and range (right) of double mutant phenotypes when two mutations can each affect one of the indicated number of different biophysical properties. Horizontal lines denote the mean of the data points. *n* = 4, 6, 4 and 1, respectively, for the groups with number of possible biophysical parameters equal to 1, 2, 3 and 4. Source data are provided as a Source data file.

We therefore considered what happens when two mutations combine and each of these mutations might have altered one of two different biophysical parameters, for example either protein stability or DNA-binding affinity. Now, even when considering expression from P_R_ as the phenotype of interest, there is always ambiguity when predicting the phenotypes of double mutants (Fig. 3a–f and Supplementary Fig. 3a–f). For example, there are now four valid phenotypic outcomes when combining two mutations if each can alter either stability or DNA binding (but not both, Fig. 3a–f). Considering expression from P_RM_ as the phenotype of interest, there are now many valid phenotypes for each double mutant when combining mutations of known effect (Fig. 3g–l and Supplementary Fig. 3g–l).

If mutations can affect any one of the four biophysical parameters, the number of possible double mutant phenotypes can be very large indeed (Fig. 3m, n and Supplementary Fig. 3m, n). For example, two mutations with known effect on P_RM_ expression can combine to produce up to 15 different double mutant phenotypes if each mutation can affect any one (and only one) of the four possible free-energy terms (Fig. 3n). Thus, when we do not know the biophysical property of a protein that is altered by each mutation, it becomes impossible to predict the phenotypes of double mutants from the phenotypes of single mutants alone.

### Biophysical pleiotropy further confounds genetic prediction

In reality, the situation can actually be worse than this because mutations can affect more than one biophysical parameter at the same time. For example, of 12 mutations changing the binding affinity of CI to DNA, half also altered the stability of the protein^27,31–33^. We define these situations when one mutation influences two or more biophysical parameters as biophysical pleiotropy.

**Fig. 4:**
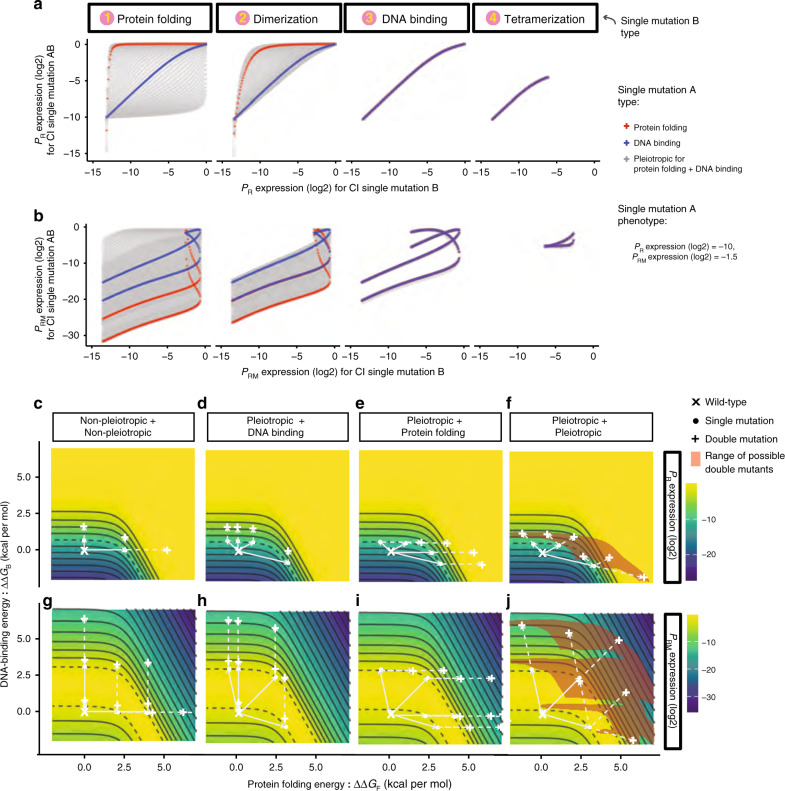
Biophysical pleiotropy further confounds phenotype prediction. **a**, **b** Double mutant phenotypes when pleiotropic mutations (Mutation A) variably affecting folding and DNA binding to give P_R_ expression = −10 (**a**) and P_RM_ expression = −1.5 (**b**) in log(2) scale are combined with different types of mutations (Mutation B). **c**–**j** Free-energy-phenotype landscapes for mutations that affect the free energy of folding (*x*-axis) and/or DNA-binding energy (*y*-axis). Phenotypic isochores are drawn with an interval of 2 in log(2) scale. A continuous range of free-energy changes can underlie an observed phenotype (dashed isochore). Combining two mutations with the same effect can result in a range of double mutant phenotypes (red shaded areas in (**f**) and (**j**)). Example double mutant outcomes are shown when neither (**c**, **g**), one (**d**, **e**, **h**, **i**) or both (**f**, **j**) mutations are pleiotropic. Source data are provided as a Source data file.

Allowing one (Fig. 4a, b, Supplementary Fig. 4) or both (Fig. 4f, j and Supplementary Fig. 4) mutations in CI to be pleiotropic and to alter two different free-energy terms results in the possible double mutant outcomes now covering a continuous range of values (Fig. 4 and Supplementary Fig. 4). Thus, when mutations are biophysically pleiotropic, we cannot predict the phenotype of a double mutant containing two mutations of precisely measured individual effects.

### Biophysical ambiguity confounds genetic prediction

To illustrate how these diverse double mutant phenotypes arise when combining pairs of mutations with identical phenotypic effects, we plot in Fig. 4c–f how the expression from P_R_ changes as a function of changes in the free energy of folding (∆∆*G*_F_) and DNA binding (∆∆*G*_B_). Non-pleiotropic mutations that only alter folding are horizontal movements in this space, mutations that only affect DNA binding are vertical movements and pleiotropic mutations are diagonal movements. All of the changes in free energy that result in the same phenotype form a phenotype isochore, for example the grey dashed curves in Fig. 4c–f represent all parameter changes that can produce a 4-fold increase (2 in log(2) scale) in P_R_ expression.

When two non-pleiotropic mutations that cause this same phenotypic change (lie on the same phenotype isochore) are combined together there are three possible combinations of free-energy changes (the two mutations alter DNA binding, folding, or one alters folding and the other binding) and two possible resulting double mutant phenotypes (Fig. 4c). When a non-pleiotropic mutation affecting DNA binding is combined with a pleiotropic mutation affecting both free-energy terms, there are many possible combinations of free-energy terms but, because of the topology of the free energy-phenotype landscape, all of the double mutants have very similar phenotypes (Fig. 4d). In contrast, when a non-pleiotropic mutation affecting folding is combined with a pleiotropic mutation, the possible double mutants do not fall on an isochore but now cover a range of possible phenotypes (Fig. 4e). Finally, when two pleiotropic mutations are combined, the possible double mutants are widely spread in the free-energy landscape (red shaded area in Fig. 4f) and take many different phenotypic values (Fig. 4f). The equivalent free-energy-phenotype landscape is plotted for P_RM_ in Fig. 4g–j and for other combinations of free-energy terms in Supplementary Fig. 4. It is both the monotonicity and symmetry of these landscapes that determines the degree of ambiguity when combining mutations.

**Fig. 5:**
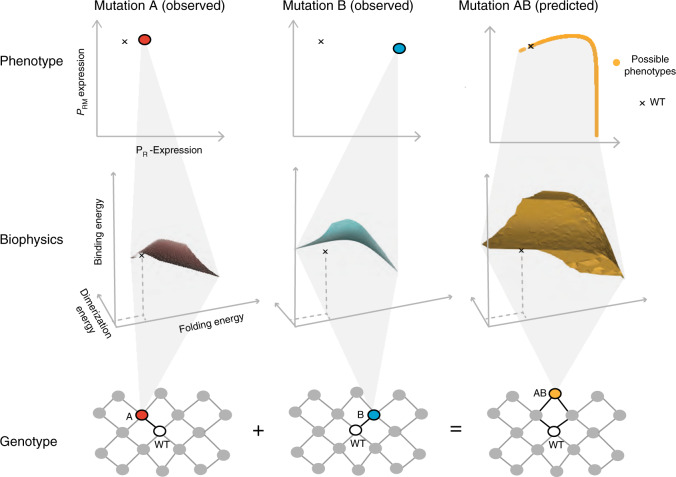
Biophysical ambiguity as a hidden layer for phenotype prediction. Mutations can alter multiple biophysical properties to cause the same observed change in a phenotype. For example, the P_R_ and P_RM_ phenotypes of mutations A and B in CI could be caused by many different changes in three free-energy terms. When these two mutations are combined, the double mutant can have a phenotype spanning nearly the entire phenotypic range, depending upon what the hidden parameter changes are in each single mutant. Source data are provided as a Source data file.

When mutations can alter three or more free-energy terms, these landscapes become difficult to visualise (Fig. 5). For example, if each mutation in CI can alter stability, DNA binding or dimerization, each mutation with a known phenotype potentially maps to any position on a surface of combinations of causal parameter changes. Combining two mutations with precisely measured phenotypic effects can combine to have phenotypes that span nearly the entire range of possible phenotype values (Fig. 5). This is because, without additional information, the actual parameter changes in the double mutant can take many values within a 3D volume of possibilities. There is now nearly complete ambiguity in the predicted phenotype of the double mutant (Fig. 5).

### Biophysical ambiguity in even simpler systems

**Fig. 6:**
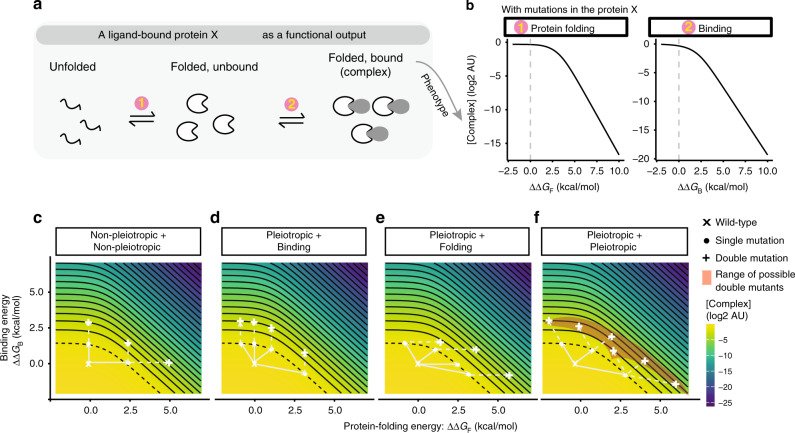
Biophysical ambiguity in a protein–protein interaction system. **a** Statistical thermodynamic model of a protein binding to a ligand. The protein X exists in three states: unfolded, folded, and folded and bound to the ligand. The partitioning of these molecules depends on the Gibbs free-energy differences between states. **b** Mutations result in additive changes in the free energy of protein folding and binding, altering the concentration of the protein–ligand complex. **c**–**f** Free-energy-phenotype landscapes for mutations that affect the free energy of folding (*x*-axis) and/or binding energy (*y*-axis). Phenotypic isochores are drawn with an interval of 1 in log(2) scale. A continuous range of free-energy changes can underlie an observed phenotype (dashed isochore). Combining two mutations with the same effect can result in a range of double mutant phenotypes (red shaded areas in (**f**)). Example double mutant outcomes are shown when neither (**c**), one (**d**, **e**) or both (**f**) mutations are pleiotropic. See also Supplementary Fig. 5. Source data are provided as a Source data file.

Finally, although gene regulation by the lambda repressor is a relatively simple biological system, we note that biophysical ambiguity also confounds the prediction of double mutant phenotypes in even simpler systems. For example, consider a protein whose only function is to bind another molecule (a ligand), with the concentration of the bound complex directly proportional to the phenotype of interest (Fig. 6a). In such a minimal system mutations can only alter protein stability or the binding affinity to the ligand. The outcome in a double mutant can still differ depending upon which free-energy terms are individually affected in each single mutant (Fig. 6b, c). Again, allowing pleiotropic mutations further thwarts the ability to predict the phenotypes of double mutants from the phenotypes of single mutants (Fig. 6d, e). Similar conclusions are obtained using a model in which a protein’s only function is to bind to itself to form a dimer (Supplementary Fig. 5). Thus, even in these most basic biological systems of a single binding reaction of a macromolecule, it is often impossible to predict what happens when single mutants of known phenotype are combined without additional measurements or inferences.

## Discussion

Taken together, our results show that, even for a simple biological system—the regulation of gene expression by a single transcription factor—it is often impossible to unambiguously predict how two mutations of known phenotypic effect will combine together to alter the same phenotype in a double mutant.

The fundamental cause of this uncertainty is the one-to-many relationship between a measured phenotype and the underlying causal changes in biophysical parameters. Mutations can affect multiple biophysical properties of a system—for example, the stability and binding affinities of proteins—and many different changes in biophysical parameters can cause the same observed change in a trait. However, the phenotype of a double mutant depends on which of these biophysical properties is actually altered in each single mutant and so can take multiple values. Pleiotropic biophysical effects and non-monotonic input–output functions create further ambiguity when predicting how mutations of known effect combine to alter a phenotype.

The extent to which biophysical ambiguities will thwart the prediction of different phenotypes will depend on the number of parameters that can be affected by mutations, their biophysical pleiotropy, and monotonicity of input–output functions. The distributions of mutational effects on multiple biophysical parameters have been quantified for very few systems, but for both the lambda repressor and other proteins, mutations frequently affect both stability^41,42^ and binding to interaction partners^41,43,44^ with biophysical pleiotropy and non-monotonic functions also common^31,35,45^. In other words, we expect biophysical ambiguity to confound phenotypic prediction in other systems including heteromeric complexes and beyond transcription factor-mediated repression.

To resolve ambiguities and accurately predict how mutations combine to alter phenotypes, additional information will always be required. Although ultimately it may be possible to predict from sequence how a particular mutation affects all the biophysical parameters of a protein, for the foreseeable future resolving ambiguities will require additional measurements to be made. High-throughput methods to quantify the effects of mutations on protein stability^42^, binding^41,44,46^ and activity^47^ will help in this endeavour, particularly when used in combination to disentangle biophysical effects. Moreover, quantifying how individual mutations interact with many other mutations in a system may allow the underlying causal changes in biophysical parameters to be inferred, at least when only two different parameters can be affected^35^. Quantifying intermediate molecular phenotypes such as protein concentrations and additional higher-level phenotypes may also be useful (e.g., quantifying expression from P_R_ is sufficient to resolve the ambiguities resulting from the non-monotonicity of the P_RM_ dose–response curve), and experimentally quantifying the dose–response curves of individual mutations can also sometimes help to distinguish mutations with different biophysical effects^48^.

However, the fundamental conclusion remains: even in this simple biological system (and in even simpler ones, Fig. 6 and Supplementary Fig. 5) it can be impossible to predict the combined effect of two mutations, even if we have perfect measurements of their individual effects on a trait. In such cases, additional information or measurements will always be required to accurately predict how genetic variants combine to alter phenotypes and cause disease.

## Methods

### Methods overview

Our model is based on Ackers’ thermodynamic model of lambda repressor binding to its operator sites (O_R_1, O_R_2 and O_R_3)^17^. Briefly, this model describes eight possible operator configuration states (c1–c8) in which the CI dimer can bind to the operators (Fig. 1b). Based on statistical thermodynamics, the downstream gene expression from promoters P_R_ and P_RM_ is determined by the probabilities of the ON and OFF *cis*-regulator configuration states^17^.

To examine CI coding mutants’ effects on gene expression from P_R_ and P_RM_ promoters, we extended Ackers’ model by including CI folding because many mutations destabilise proteins^22–25^. Destabilising mutations will decrease the fraction of the folded functional protein, and thus change gene expression from the downstream P_R_ or P_RM_ promoter. In other words, compared to Ackers’ model, we have one more protein state—CI unfolded state CI_(U)_ and the corresponding additional parameter—protein-folding energy ∆*G*_(F)_ (Supplementary Tables 1 and 2). The rest of our model is the same as Ackers’ model. We consider the system as a single equilibrium, i.e. protein folding and dimerization are coupled reactions.

Below are the details of the model, which follow simple statistical thermodynamics.

### CI configuration states

The total CI (CI_(Total)_) molecule amount is the sum of all the CI molecules in the 10 different possible states as shown in Eq. (1). These different states include unfolded CI_(U)_, folded monomer CI_(M)_, free dimer CI_2_ and seven operator-bound CI dimer states (Fig. 1b and Supplementary Table 1). The unit of molecule amount per cell is M in all the equations in our model.

1 $${\mathrm{CI}}_{({\mathrm{Total}})} = {\mathrm{CI}}_{({\mathrm{U}})} + {\mathrm{CI}}_{({\mathrm{M}})} + 2 \cdot {\mathrm{CI}}_2 + 2 \cdot {\mathrm{OR}}_{({\mathrm{Total}})}\mathop {\sum}\limits_{i = 2}^7 {\left( {k \cdot f_i} \right)}.$$

Above, ${\mathrm{OR}}_{({\mathrm{Total}})}$ is the molecule amount of the operators, *f*_*i*_ is the relative probability that each of the seven *cis*-configuration states where CI is bound to operators occurs in relation to the not-bound state. *i* is the index for each *cis*-configuration state, and *k* is the number of CI dimers in the corresponding *cis*-configuration state (Supplementary Table 1). The amount of CI molecule for each operator-bound state is calculated based on the statistical thermodynamics but also multiplying the number of CI dimers (*k*) in each state and a factor 2 to account for two molecules for each dimer (Supplementary Table 1).

All the parameters in the model for wild-type CI are taken from literature (Supplementary Table 2).

### Equilibrium between CI unfolded and folded monomer states

CI monomer folds in a simple folded CI_(M)_ and unfolded CI_(U)_ two-state fashion^49^ that can be described as in the equation below:

2 $$\frac{{{\mathrm{CI}}_{({\mathrm{M}})}}}{{{\mathrm{CI}}_{({\mathrm{U}})}}} = \exp \left( {\frac{{ - {\Delta} G_{\mathrm{F}}}}{{RT}}} \right).$$

Δ*G*_F_ is the free-energy difference between the folded monomer and unfolded states of CI molecule. *R* is the gas constant (*R* = 1.98 × 10^−3^ kcal per M) and *T* is the absolute temperature for 37 °C (310.15 Kelvin).

### Equilibrium between folded CI monomer and free dimer states

3 $$\frac{{{\mathrm{CI}}_2}}{{{\mathrm{CI}}_{({\mathrm{M}})}^2}} = \exp \left( {\frac{{ - {\Delta} G_{\mathrm{D}}}}{{RT}}} \right).$$

### Equilibrium between free CI dimer and operator-bound states

We use Ackers’ model to describe these relationships. Briefly, the likelihood of each configuration state (c1–c8 based on the *cis*-regulatory state) is a function of the binding energies and the free CI protein dimer concentration.

The probability that each of the eight *cis*-configuration states $\left( {f_i} \right)$ occurs is:

4 $$f_i = \frac{{{\mathrm{exp}}\left( {\frac{{ - {\Delta} G_i}}{{RT}}} \right){\mathrm{CI}}_2^k}}{{\mathop {\sum }\nolimits_i {\mathrm{exp}}\left( {\frac{{ - {\Delta} G_i}}{{RT}}} \right){\mathrm{CI}}_2^k}}.$$

Where ${\Delta} G_i$ is the total free energy of lambda repressor dimers in the respective *cis*-configuration state *i* ∈ [1, 8] (Supplementary Table 1, where Δ*G* is free energy, with Δ*G*_T_ referring to the cooperation energy for two dimers binding to the adjacent operator sites); the exponent *k* ∈ [0,1,2] is the total number of the lambda repressor dimers in the corresponding *cis*-configuration state *i*. As stated earlier, all the parameters are kept as originally described in Ackers’ model (Supplementary Table 2).

### CI distribution based on statistical thermodynamics

By combining Eqs. (1)–(4), we can describe the total expression level of CI_(Total)_ as a function of CI free dimer concentration and Gibbs free energies:

5 $$\begin{array}{l}{\mathrm{CI}}_{({\mathrm{Total}})} = \exp \left( {\frac{{{\Delta} G_{\mathrm{D}} + {\Delta} G_{\mathrm{F}}}}{{RT}}} \right){\mathrm{CI}}_2^{0.5} + 2{\mathrm{CI}}_2 \\ + \frac{{2{\mathrm{OR}}\left( {\mathop {\sum }\nolimits_{i = 2}^4 \exp \left( {\frac{{ - {\Delta} G_{\mathrm{i}}}}{{RT}}} \right){\mathrm{CI}}_2 + 2 \times \mathop {\sum }\nolimits_{i = 5}^7 \exp \left( {\frac{{ - {\Delta} G_i}}{{RT}}} \right){\mathrm{CI}}_2^2 + 3\exp \left( {\frac{{ - {\Delta} G_8}}{{RT}}} \right){\mathrm{CI}}_2^3} \right)}}{{\mathop {\sum }\nolimits_{i = 2}^4 \exp \left( {\frac{{ - {\Delta} G_i}}{{RT}}} \right){\mathrm{CI}}_2 + \mathop {\sum }\nolimits_{i = 5}^7 \exp \left( {\frac{{ - {\Delta} G_i}}{{RT}}} \right){\mathrm{CI}}_2^2 + \exp \left( {\frac{{ - {\Delta} G_8}}{{RT}}} \right){\mathrm{CI}}_2^3}}\end{array}.$$

### Probability of P_R_—ON

CI represses expression from the P_R_ promoter by binding to the operator sites that overlap with the RNA polymerase sigma factor binding site (Fig. 1b)^17^. Based on Ackers’ model, two out of the eight *cis*-configuration states fail to repress gene expression from P_R_—when CI is not bound to any operators (c1) and when CI only binds to the low-affinity O_R_3 (c2) (Fig. 1b, Supplementary Table 1). Therefore, the probability of the P_R_ promoter to be active (*P*_pr_) is the sum of the probabilities of the two configuration states in which promoter P_R_ is not repressed $\left( {\mathop {\sum }\nolimits_{i = \left\{ {1,2} \right\}} f_i} \right)$, as shown in Eq. (6)^17^.

6 $$P_{{\mathrm{pr}}} = f_1 + f_2 = \frac{{\exp \left( {\frac{{ - {\Delta} G_1}}{{RT}}} \right){\mathrm{CI}}_2^0 + \exp \left( {\frac{{ - {\Delta} G_2}}{{RT}}} \right){\mathrm{CI}}_2^1}}{{\mathop {\sum }\nolimits_{i = 1}^8 \left( {\exp \left( {\frac{{ - {\Delta} G_i}}{{RT}}} \right){\mathrm{CI}}_2^k} \right)}}.$$

### Probability of P_RM_—ON

CI not only suppresses P_R_ promoter but also activates or suppresses the divergently transcribed P_RM_ promoter in response to changes in the CI concentration in the cell (Fig. 1c)^10,50^. When CI is present and binds to O_R_2, it activates the P_RM_ promoter, while binding to O_R_1 per se does not have any effects on P_RM_ activity^10,16^. On the contrary, once CI binds to the low-affinity O_R_3, it blocks the access of RNA polymerase sigma factor, repressing expression from P_RM_^51^. Therefore, gene expression from P_RM_ is activated only when CI is bound to O_R_2 and not bound to O_R_3 (corresponding to the two *cis*-configuration states: c3 and c7) (Fig. 1b and Supplementary Table 1). Using Ackers’ model and Eq. (4)^17^, we describe the probability that the P_RM_ promoter is activated as follows:

7 $$P_{{\mathrm{prm}}} = f_3 + f_7 = \frac{{\exp \left( {\frac{{ - {\Delta} G_3}}{{RT}}} \right){\mathrm{CI}}_2^1 + \exp \left( {\frac{{ - {\Delta} G_7}}{{RT}}} \right){\mathrm{CI}}_2^2}}{{\mathop {\sum }\nolimits_{i = 1}^8 \left( {\exp \left( {\frac{{ - {\Delta} G_i}}{{RT}}} \right){\mathrm{CI}}_2^k} \right)}}.$$

### Calculating free dimer concentration

As seen from Eq. (5), we can easily calculate CI_(Total)_ from CI_2_ for a given set of free energies but not CI_2_ from CI_(Total)_. Therefore, we performed a parameter search for CI_2_ values with each set of known biophysical parameters (∆*G* values) that minimizes the absolute differences between the provided CI_(Total)_ value and CI_(Total)_ calculated based on Eq. (5). The Optimize^52^ function in R was used for the parameter search, with the tol parameter set to 1e−23. We refer to this process using Eq. (8), where ${\Delta} G_{\mathrm{s}}$ are all the Gibbs free energies of the system.

8 $${\mathrm{CI}}_2 = f\left( {{\mathrm{CI}}_{({\mathrm{Total}})},{\Delta} G_{\mathrm{s}}} \right).$$

### Biophysical changes to phenotypes

The probabilities of the two promoters’ ON-states as phenotypes can be calculated using a set of biophysical parameters (free energies) and CI_(Total)_. We call this process a Forward Function (see Code availability). This function is composed of two steps: (1) parameter search for CI_2_ for the given CI as described in the previous section (Calculating free dimer concentration) using Eq. (8); (2) calculating P_PR_ and P_PRM_ based on Eqs. (6) and (7).

### Phenotypes to free energy for non-pleiotropic mutations

Mutations in the CI protein can affect protein-folding energy (Δ*G*_F_), dimerization energy (Δ*G*_D_), binding energy to the operator sites (Δ*G*_OR1–OR3_) and tetramerization energy (Δ*G*_T_) at the biophysical level. We assume that mutations in CI that alter the free energy of DNA binding do so by the same magnitude for all three operators (ΔΔ*G*_B_ = ΔΔ*G*_OR1_ = ΔΔ*G*_OR2_ = ΔΔ*G*_OR3_). To calculate only one biophysical change that can lead to the phenotype, we reversed the Forward Function described in the previous section. The Reverse Function for both P_PR_ and P_PRM_ is composed of two sub-functions. The first sub-function is the above-mentioned Forward Function, which calculates phenotypes from biophysical changes. This function is written in the form of *y* = f(*x*), where *y* is the phenotype and *x* is a set of biophysical parameters including the total expression level of CI. The second sub-function is an Inverse Function that finds all roots for an equation in the form of *y* – f(*x*) = 0. A root-finding process is performed using the uniroot.all function in the R package rootSolve^53^. Specifically, for each perturbation of biophysical parameter (∆∆*G*), we looked for all the roots within a range of −2–10 kcal per mol, and returned the ∆∆*G* values that produce the phenotypes while the other biophysical parameters are not perturbed.

Mutational effects are modelled at a fixed expression level of CI $( {{\mathrm{CI}}_{({\mathrm{Total}})} = 8.4{\mathrm{e}} - 7{\mathrm{M}}} )$ that corresponds to ~99% repression of the P_R_ promoter and the CI concentration in a lysogen^17,19^. To calculate changes in the biophysical parameters for single mutants with known effects on expression from P_R_ or P_RM_, we first generated 136 evenly spaced phenotypes (with an interval of 0.1 in log(2) scale from −13.5 to 0). Then, for a given phenotype, we calculated corresponding changes in any of the four free-energy terms (biophysical parameters), each time allowing only one biophysical parameter to change using the Reverse Function explained in in the previous paragraph.

### Phenotypes to free energy for pleiotropic mutations

For any given phenotype, we systematically searched for combinations of biophysical changes that can produce the phenotype. Taking a pleiotropic mutation affecting both protein-folding energy (Δ*G*_F_) and DNA-binding energy (Δ*G*_B_) as an example, we first generated a fixed range of ΔΔ*G*_F_ (−1 to 5 kcal per mol with an interval of 0.05 kcal per mol). Then, for each ΔΔ*G*_F_, we calculated ΔΔ*G*_B_ that produces the given phenotype using the Reverse Function as described for non-pleiotropic mutations. For mutations affecting three biophysical parameters (protein-folding energy Δ*G*_F_, dimerization energy Δ*G*_D_ and DNA-binding energy Δ*G*_B_), we first generated all possible two-way combinations of ΔΔ*G*_F_ and ΔΔ*G*_D_, each from defined ranges of −1 to 5 kcal per mol with an interval of 0.05 kcal per mol. For each combination of ΔΔ*G*_F_ and ΔΔ*G*_D_ with the given phenotype, we calculated ΔΔ*G*_B_, using the Reverse Function as described for non-pleiotropic mutations.

### Double mutant phenotypes from single mutants’ phenotypes

For each double mutant, we simply added the changes in the free energies of both single mutants to the corresponding wild-type free energy. Then, we used the updated parameters to calculate the downstream phenotypes based on the Forward Function explained in the section of Phenotypes to free energy for non-pleiotropic mutations. Double mutants’ phenotypes are rounded to 2 decimal places in log(2) scale in order to avoid counting phenotypes with very similar values as different phenotypes.

### Thermodynamic model of simple protein interactions

We considered the protein of interest (that is mutated) to be in three different configuration states: (1) unfolded, (2) folded, and (3) folded and bound (or dimer) (Fig. 6a and Supplementary Fig. 5a). The steady-state equilibrium is in the same format as shown for CI protein in Eqs. (2) and (3). When protein binds to a substrate instead of to itself, it follows Eq. (9).

9 $$\frac{{[{\mathrm{Complex}}]}}{{\left[ {{\mathrm{ProteinX}}} \right] \cdot \left[ {{\mathrm{Ligand}}} \right]}} = \exp \left( {\frac{{ - {\Delta} G_{\mathrm{B}}}}{{RT}}} \right).$$

Above, [complex] is the concentration of the bound Protein X to its ligand (or substrate molecule). The parameters we used in the model for Figs. 6 and S5 are ∆*G*_F, WT_ = −1 kcal per mol; ∆*G*_B (or D), WT_ = −2 kcal per mol. [Protein X]:[Ligand] = 1:1.

### 3D visualisation of CI bound to O_R_1–3

The 3D structure of CI bound to O_R_1–3 was generated based on PDB structure 3bdn, using YASARA software (v 19.7.20).

### Reporting summary

Further information on research design is available in the Nature Research Reporting Summary linked to this article.

## Supplementary information

Supplementary Information

Peer Review File

Reporting Summary

## Data Availability

All data supporting this work are provided within the paper, the supplementary information and the source data file. Source data are provided with this paper.

## Source data

Source Data

## Code availability

Scripts are publicly available from https://github.com/lehner-lab/Biophysical_Ambiguity. Source data are provided with this paper.
