# Supplementary material for: Biophysical ambiguities prevent accurate genetic prediction
Source: Nat Commun. 2020 Oct 1;11:4923. doi: 10.1038/s41467-020-18694-0 (PMC7529754; doi:10.1038/s41467-020-18694-0)
Supplement: Supplementary file 1 — Supplementary Information [file 41467_2020_18694_MOESM1_ESM.pdf]

Supplementary Information

Biophysical ambiguities prevent accurate genetic prediction

Li et al.

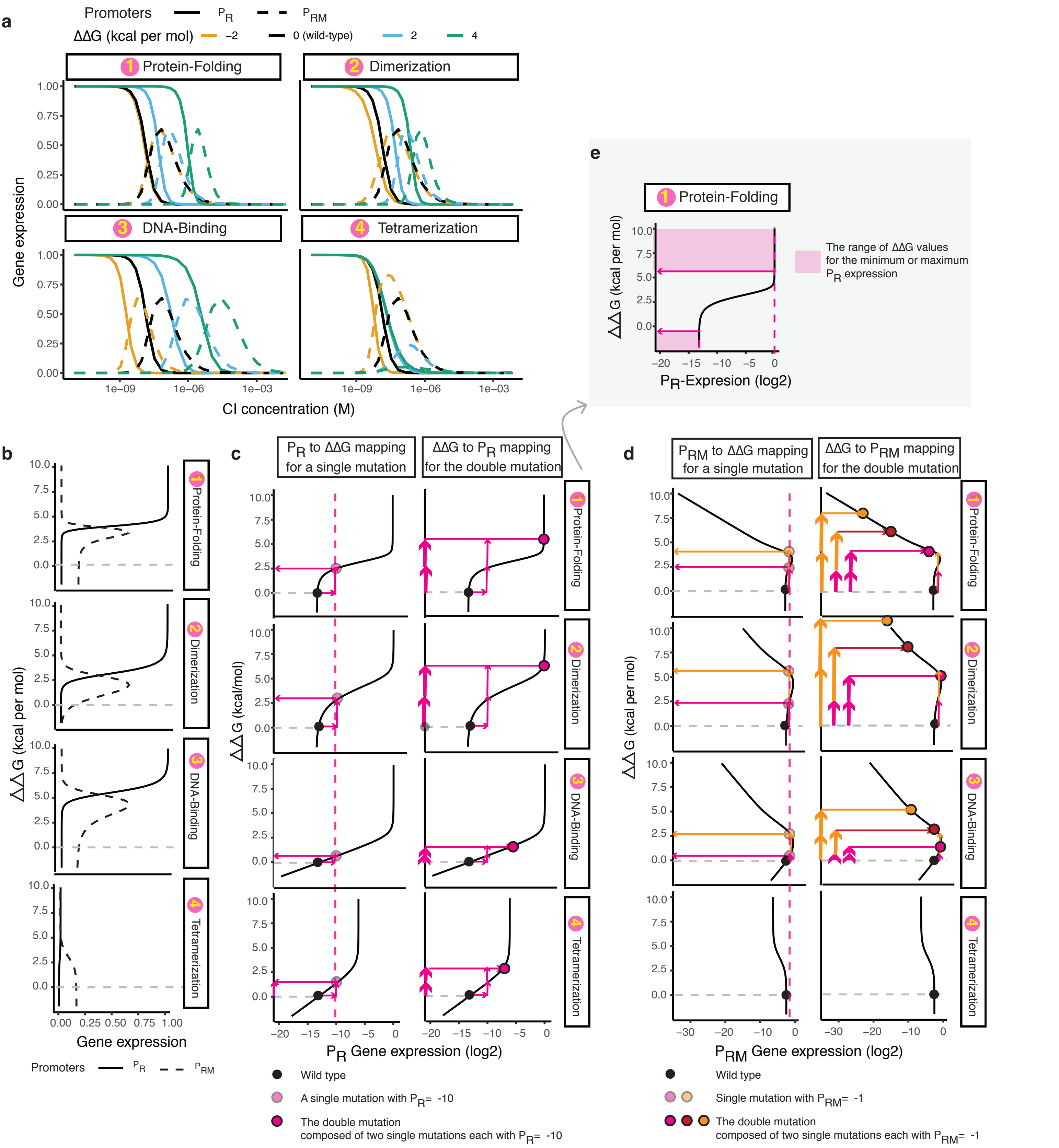

Supplementary Figure 1. Relationships between biophysical parameters and phenotypes. (a) Dose-response curves of the P_R and P_{RM} promoters with altered biophysical parameters. **(b)** P_R and P_{RM} phenotype - Free energy functions for mutations affecting the free energy of protein-folding, dimerization, DNA-binding, and tetramerization with the phenotypes. **(c, d)** Scheme for mapping P_R **(c)** and P_{RM} **(d)** phenotypes to biophysical parameters for single mutations (left panels) and from biophysical parameters to phenotypes for double mutations (right panels). Phenotypes are shown in log(2) scale. **(e)** Ambiguous mapping of phenotype to free energy changes due to measurement uncertainty when mutant phenotypes are close to minimum or maximum possible values. Source data are provided as a Source Data file.

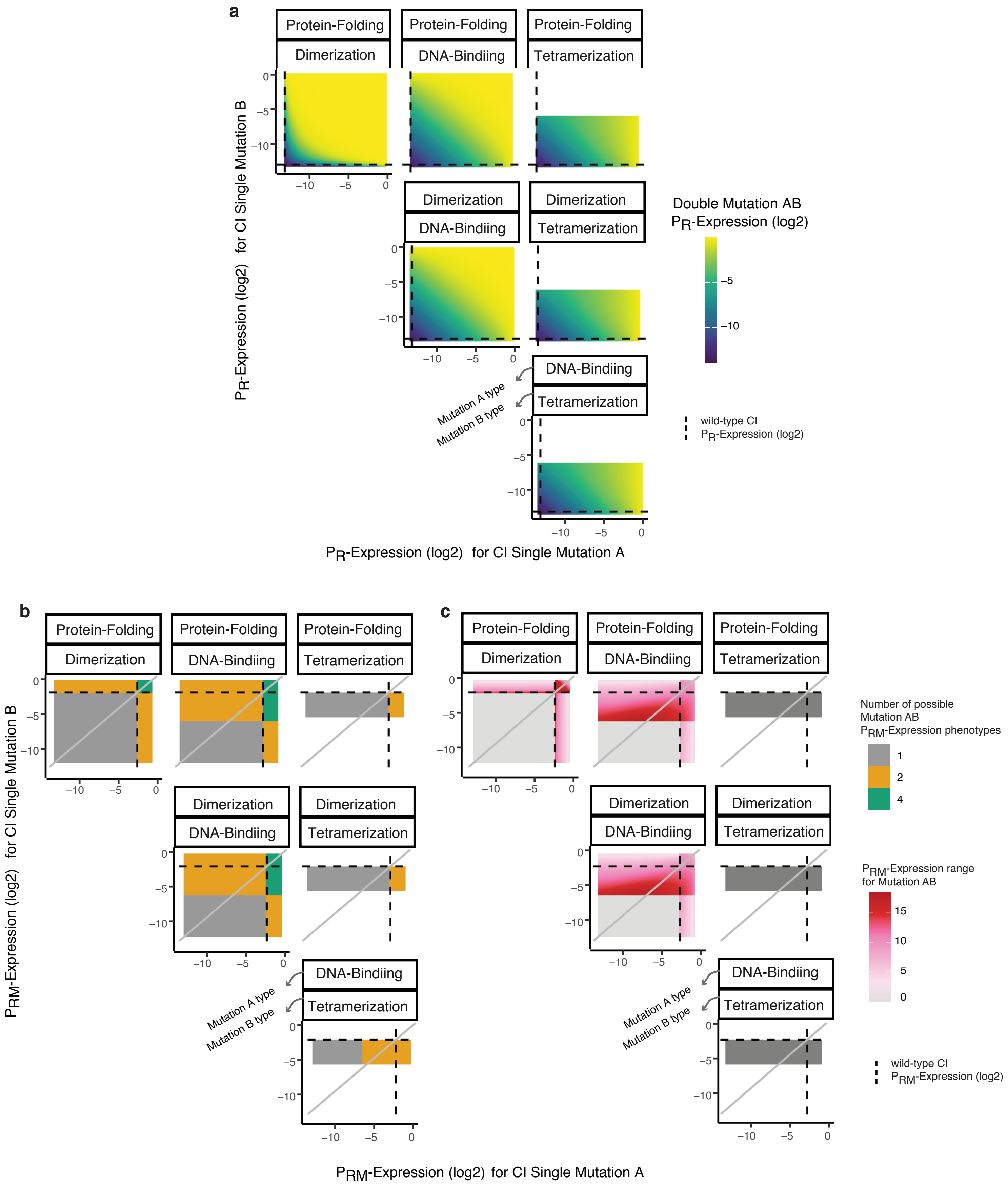

Supplementary Figure 2. Combining mutations affecting two different biophysical parameters. (a) Double mutant P_R phenotypes. **(b, c)** Number **(b)** and range **(c)** of possible double mutant P_{RM} phenotypes. Source data are provided as a Source Data file.

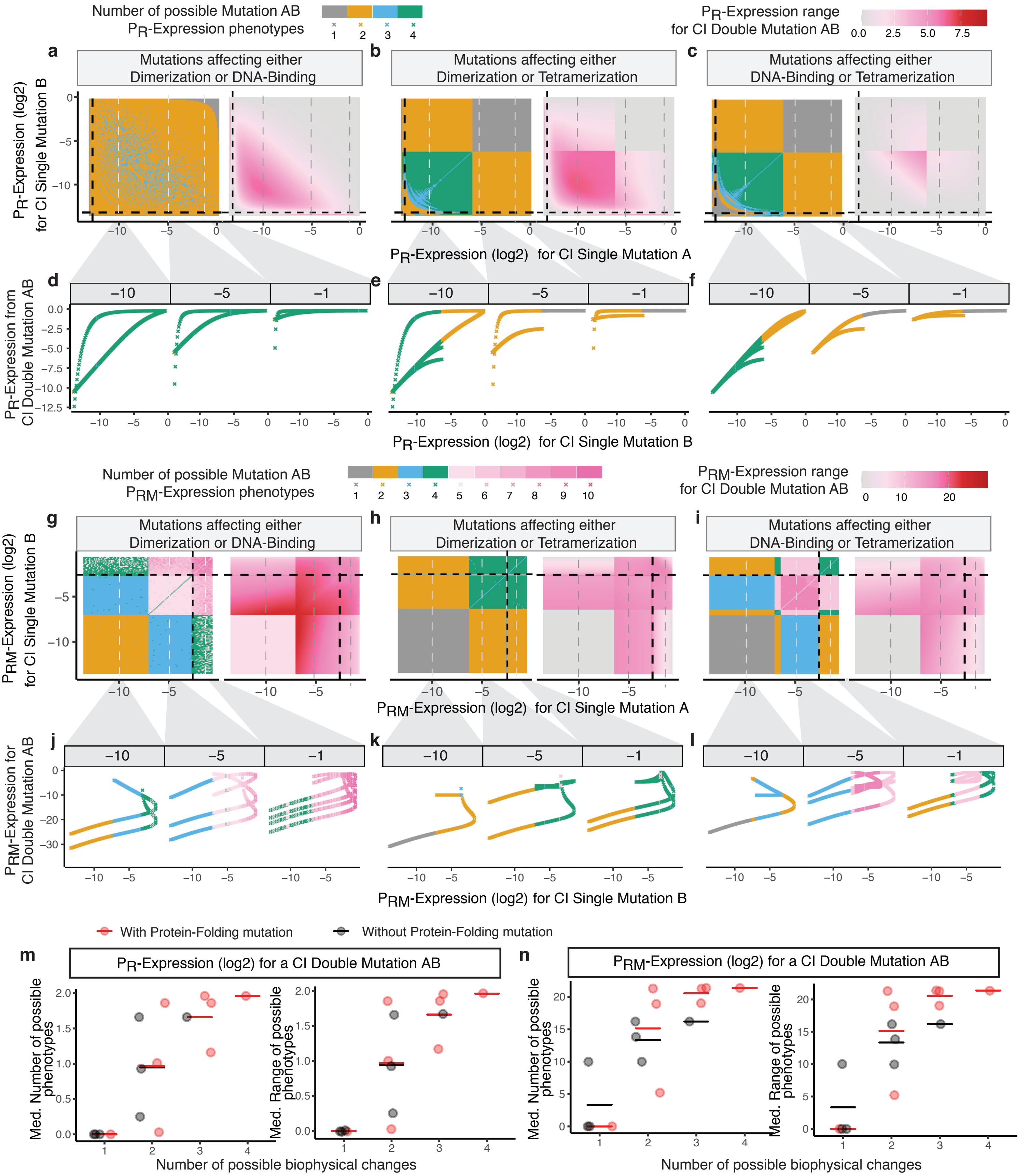

Supplementary Figure 3. Biophysical ambiguity confounds phenotype prediction. (**a** – **c**) Double mutant P_R phenotypes when two single mutants can affect either of the two indicated biophysical parameters (but not both). Number of possible double mutant P_R phenotypes (left panels) and range of possible double mutant P_R phenotypes (right panels) are shown for each combination of single mutant phenotypes. (**d** – **f**) Examples showing how a mutation with a known phenotype combines with other mutations leading to 1 to 4 possible double mutant PR phenotypes. (**g** – **i**) Double mutant P_{RM} expression when two single mutants are allowed to affect either of the two indicated biophysical parameters. Number (left panels) and range of possible double mutant P_{RM} phenotypes (right panels) are shown for each combination of single mutant P_{RM} phenotypes. (**j** – **l**) Examples showing how a mutation with a known P_{RM} phenotype combines with other mutations, leading to many different possible double mutant P_{RM} phenotypes. (**m**, **n**) The uncertainty of possible double mutant P_R (**m**) and P_{RM} (**n**) phenotypes increases with increasing biophysical ambiguity. Each data point represents the median number (left panels) and the median range (right panels) of double mutant phenotypes for a given combination of biophysical types. Horizontal lines denote the mean of the data points. n= 4, 6, 4 and 1 respectively for the groups with number of possible biophysical parameters equal to 1, 2, 3 and 4. Source data are provided as a Source Data file.

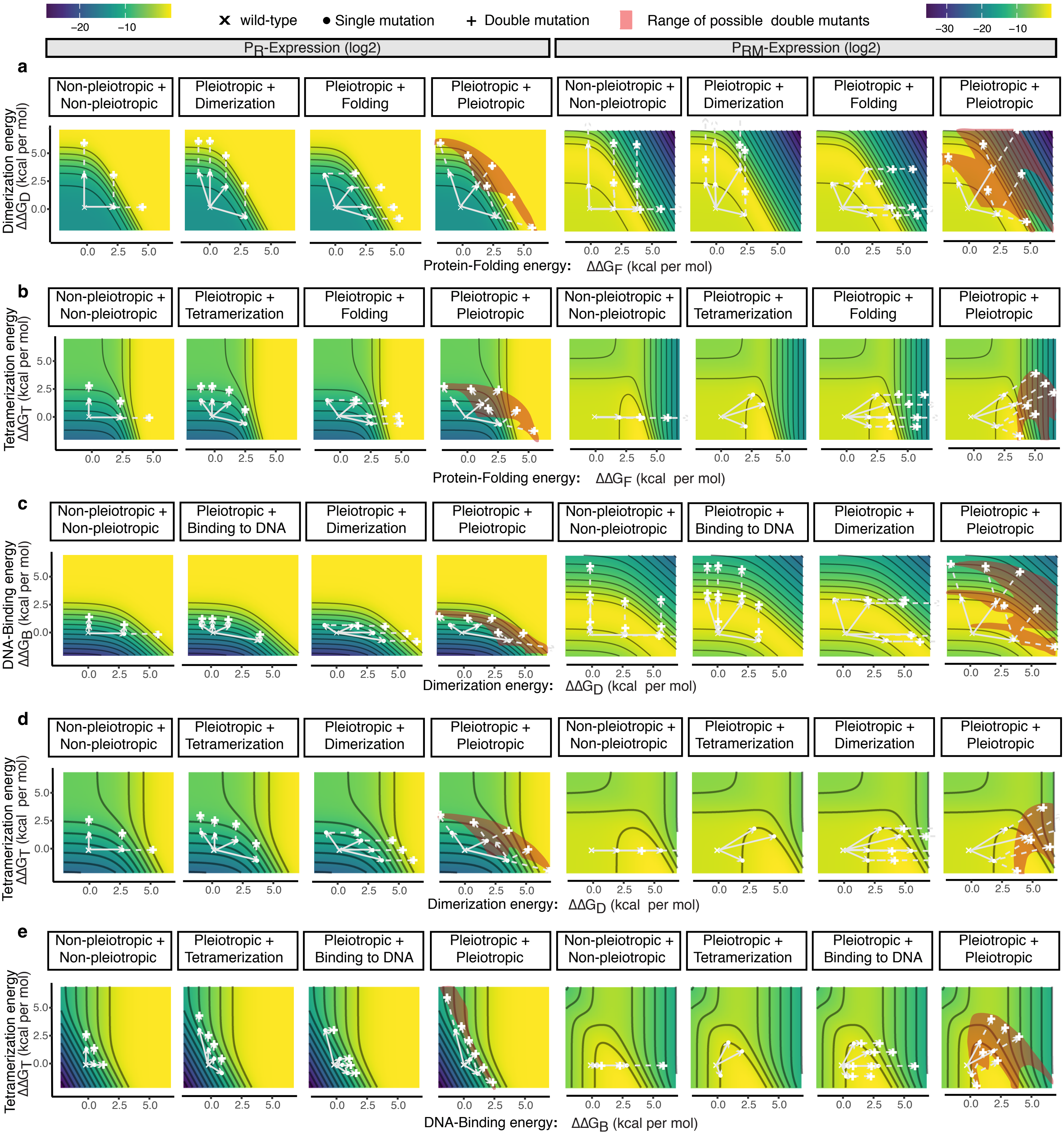

Supplementary Figure 4. Pleiotropic mutations confound phenotype predictions. Free energy-phenotype landscapes when mutations can affect the free energy of (**a**) protein-folding and DNA-binding, (**b**) protein-folding and dimerization, (**c**) protein-folding and tetramerization, (**d**) dimerization and DNA-binding, (**e**) dimerization and tetramerization, (**f**) DNA-binding and tetramerization. The phenotypic isochores are drawn with a phenotypic interval of 2 in log(2) scale. The red-shaded area marks the range of possible double mutant phenotypes for P_R (left four panels) and P_{RM} (right four panels) when two mutations both with the same phenotype are combined. Examples of mutations combined with tetramerization-only mutations are missing in the panels (**c**, **e**, and **f**) because, when a mutation only affects tetramerization it cannot result in this P_{RM} phenotype (PRM=-2). Source data are provided as a Source Data file.

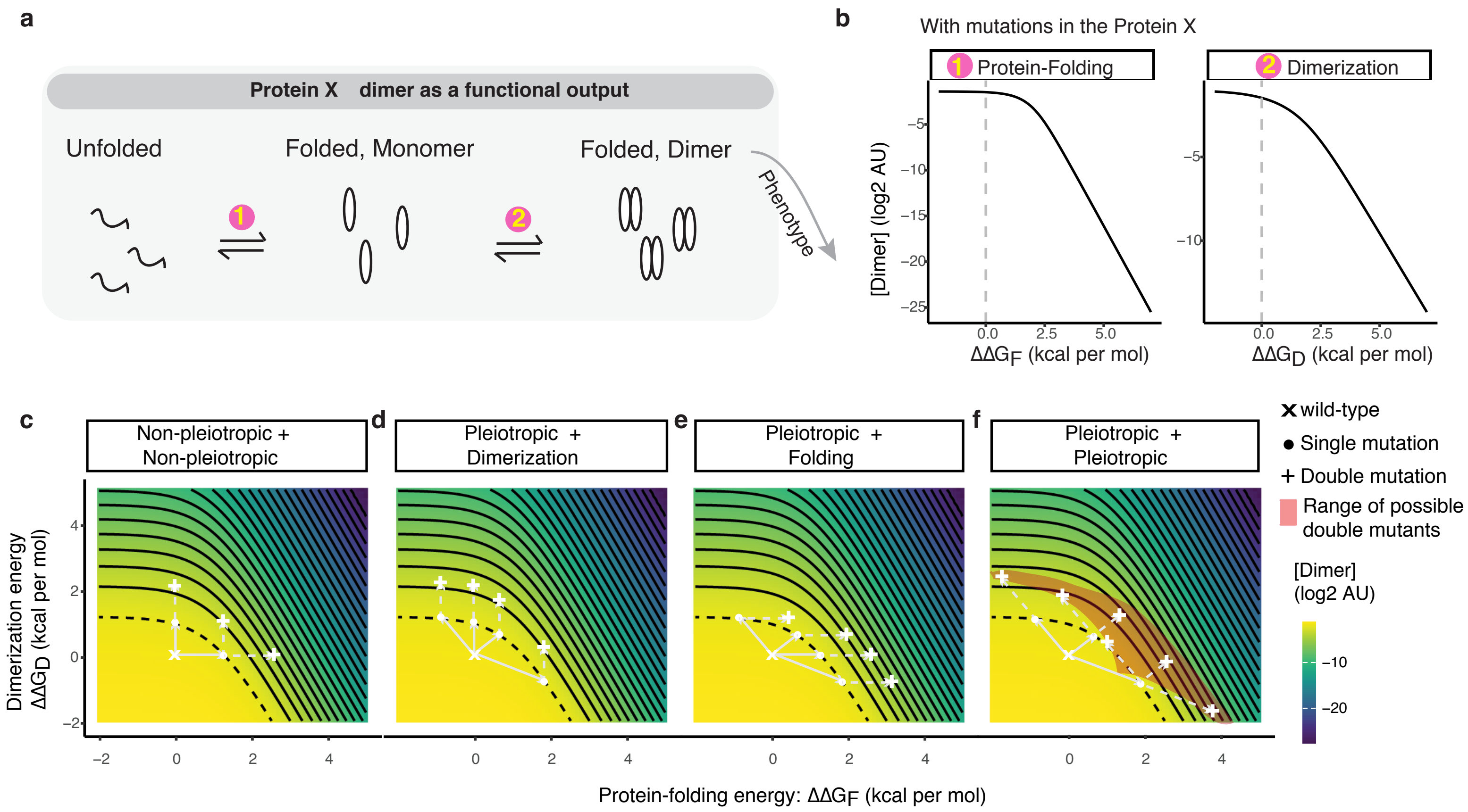

Supplementary Figure 5. Biophysical ambiguity in a protein dimer system. (**a**) Statistical thermodynamic model of protein dimerization. Protein X exists as 3 states: unfolded, folded monomer and folded dimer. The partitioning of these molecules depends on Gibbs free energy differences between states. (**b**) Mutations result in additive changes in the free energy of protein folding and dimerization, altering the protein dimer concentration. (**c** – **f**) Free energy-phenotype landscapes for mutations that affect the free energy of folding (x-axis) and/or dimerization energy (y-axis). Phenotypic isochores are drawn with an interval of 1 in log(2) scale. A continuous range of free energy changes can underlie an observed phenotype (dashed isochore). Combining two mutations with the same effect can result in a range of double mutant phenotypes (red shaded areas in **f**). Example double mutant outcomes are shown when neither (**c**), one (**d**, **e**) or both (**f**) mutations are pleiotropic. Source data are provided as a Source Data file.

Supplementary Table 1. Statistical thermodynamics of CI regulatory system

Cis-states	CI states	# CI_2 bound	P_R	P_{RM}	Statistical thermodynamic transition states	Energy-terms between the statistical thermodynamic transition states	Relative distribution of cis-regulatory element states based on the statistical thermodynamics	CI Relative molecule numbers, based on the statistical thermodynamics
	Unfolded $CI_{(U)}$				$CI_{(U)} / CI_{(M)}$	$-\Delta G_F$		$\exp\left(\frac{0.5\Delta G_D + \Delta G_F}{RT}\right) CI_2^{0.5}$
	Monomer $CI_{(M)}$				$CI_{(M)}^2 / CI_2$	$-\Delta G_D$		$\exp\left(\frac{0.5\Delta G_D}{RT}\right) CI_2^{0.5}$
**c1**	Free Dimer CI_2	0	ON	OFF	Reference	**0**	$f_1 = \exp\left(\frac{0}{RT}\right) CI_2^{0}$	$2CI_2$
**c2**	Bound to OR3	1	ON	OFF	$c2 / (CI_2 \cdot OR)$	$\Delta G_2 = \Delta G_{OR3}$	$f_2 = \exp\left(\frac{-\Delta G_2}{RT}\right) CI_2^{1}$	$1 \cdot 2\mathrm{OR} \cdot \exp\left(\frac{-\Delta G_2}{RT}\right) CI_2^{1}$
**c3**	Bound to OR2	1	OFF	ON	$c3 / (CI_2 \cdot OR)$	$\Delta G_3 = \Delta G_{OR2}$	$f_3 = \exp\left(\frac{-\Delta G_3}{RT}\right) CI_2^{1}$	$1 \cdot 2\mathrm{OR} \cdot \exp\left(\frac{-\Delta G_3}{RT}\right) CI_2^{1}$
**c4**	Bound to OR1	1	OFF	OFF	$c4 / (CI_2 \cdot OR)$	$\Delta G_4 = \Delta G_{OR1}$	$f_4 = \exp\left(\frac{-\Delta G_4}{RT}\right) CI_2^{1}$	$1 \cdot 2\mathrm{OR} \cdot \exp\left(\frac{-\Delta G_4}{RT}\right) CI_2^{1}$
**c5**	Bound to OR1, OR3	2	OFF	OFF	$c5 / (CI_2^{2} \cdot OR)$	$\Delta G_5 = \Delta G_{OR1} + \Delta G_{OR3}$	$f_5 = \exp\left(\frac{-\Delta G_5}{RT}\right) CI_2^{2}$	$2 \cdot 2\mathrm{OR} \cdot \exp\left(\frac{-\Delta G_5}{RT}\right) CI_2^{2}$
**c6**	Bound to OR2, OR3	2	OFF	OFF	$c6 / (CI_2^{2} \cdot OR)$	$\Delta G_6 = \Delta G_{OR2} + \Delta G_{OR3} + \Delta G_T$	$f_6 = \exp\left(\frac{-\Delta G_6}{RT}\right) CI_2^{2}$	$2 \cdot 2\mathrm{OR} \cdot \exp\left(\frac{-\Delta G_6}{RT}\right) CI_2^{2}$
**c7**	Bound to OR1, OR2	2	OFF	ON	$c7 / (CI_2^{2} \cdot OR)$	$\Delta G_7 = \Delta G_{OR1} + \Delta G_{OR2} + \Delta G_T$	$f_7 = \exp\left(\frac{-\Delta G_7}{RT}\right) CI_2^{2}$	$2 \cdot 2\mathrm{OR} \cdot \exp\left(\frac{-\Delta G_7}{RT}\right) CI_2^{2}$
**c8**	Bound to OR1, OR2, OR3	3	OFF	OFF	$c8 / (CI_2^{3} \cdot OR)$	$\Delta G_8 = \Delta G_{OR1} + \Delta G_{OR2} + \Delta G_{OR3} + \Delta G_T$	$f_8 = \exp\left(\frac{-\Delta G_8}{RT}\right) CI_2^{3}$	$3 \cdot 2\mathrm{OR} \cdot \exp\left(\frac{-\Delta G_8}{RT}\right) CI_2^{3}$
**Sum**							$\sum_{i\{1:8\}} f_i$	$CI_{(Total)}$

Red colour highlights values derived from the information that each CI dimer is composed of 2 CI monomers; light blue highlights number of CI dimer molecules bound to OR1-3; green highlights promoter states being ON.

Supplementary Table 2. Parameters

	Value	Unit	Reference
ΔG_F	-1	kcal per mol	1, 2
ΔG_D	-10.886	kcal per mol	2, 3
ΔG_{OR1}	-11.7	kcal per mol	2, 3
ΔG_{OR2}	-10.1	kcal per mol	2, 3
ΔG_{OR3}	-10.1	kcal per mol	2, 3
ΔG_T	-2	kcal per mol	2, 3
OR	1e-9	M	2, 3
$CI_{(Total)}$	8.4e-7	M	2

Supplementary References

1. Huang, G. S. & Oas, T. G. Structure and Stability of Monomeric .lambda. Repressor: NMR Evidence for Two-State Folding. *Biochemistry* **34**, 3884–3892 (1995).
2. Li, X., Lalic, J., Baeza-Centurion, P., Dhar, R. & Lehner, B. Changes in gene expression predictably shift and switch genetic interactions. *Nat. Commun.* **10**, 3886 (2019).
3. Ackers, G. K., Johnson, A. D. & Shea, M. A. Quantitative model for gene regulation by lambda phage repressor. *Proc. Natl. Acad. Sci. U. S. A.* **79**, 1129–33 (1982).
