# Supplementary material for: Biophysical ambiguities prevent accurate genetic prediction
Source: Nat Commun. 2020 Oct 1;11:4923. doi: 10.1038/s41467-020-18694-0 (PMC7529754; doi:10.1038/s41467-020-18694-0)
Supplement: Supplementary file 2 — Peer Review File [file 41467_2020_18694_MOESM2_ESM.docx]

Peer Review Information

**Journal:** Nature Communications

**Manuscript title:** Biophysical ambiguities prevent accurate genetic prediction

**Corresponding author name(s):** Prof Ben Lehner

**Editorial notes:**

**Transferred manuscripts**

This document only contains reviewer comments and rebuttal letters for versions considered at *Nature Communications*.

**Reviewer comments & decisions:**

**Reviewer comments, first version:**

Reviewer #1 (Remarks to the Author):

Thank you for the opportunity to review “Biochemical ambiguities prevent accurate genetic prediction” by Li and Lehner. This manuscript reports on the challenges to predict phenotype resulting from a combination of mutations by only relying on their individual effects without additional phenotypic measurements. Authors use phage lambda repressor (CI), a well-studied protein and a model for gene regulation, to computationally model the expression of its two target genes using a thermodynamic model. They tested how mutations in CI that exert different biochemical effects, specifically its binding affinity or protein stability, affect target gene expression. They show that one-to-many mapping between phenotypes, made it impossible to predict how two mutations in CI combine without incorporating biochemical measurements that resolve ‘biochemical ambiguities’.

This study investigates how predictable the effect of combinations of mutations is on cellular phenotypes. This addresses an important question in the field of genetic interactions and provides novel insight about the difficulty to make such predictions based on sole phenotypes of single mutants without other measurements, in this case biochemical. Overall, this is a really nice paper.

Specific comments:

1. The introduction should describe Acker’s statistical thermodynamic model since the manuscript is rooted in this model.

2. In Fig. 1D, it is not clear what the dotted vertical black line is supposed to represent.

3. In Fig. 4A-B single mutation is denoted as ‘x’ in the figure legend however the figure itself uses ‘+’. Authors should be consistent with their notation.

4. In Fig. 4C-J, I had a difficult time differentiating the grey ‘x’ from everything else in the plot.

5. Authors should incorporate into the discussion, what would be the expected behaviour of heteromers and multisubunit protein complexes given their findings about CI homomers.


Reviewer #2 (Remarks to the Author):

In this paper, the Authors demonstrate that it can be impossible to predict the joint effect of two mutations in one genetic background from their individual effects observed in isolation. They use the classical lambda repressor system that is very well understood, to make this point explicitly.

To some extent, the claim is trivial - mathematically, not all functions are injective; statistically, marginal distributions of two variables are not generally sufficient to reconstruct their full joint distribution; in genetics, interactions have been appreciated for over a century. The novelty is demonstrating that knowing the mechanism does not rescue this ability, even in the simplest known two-stage systems. 

In a sense, the manuscript raises more questions than it answers, but perhaps this is not a bad thing. The point it does attempt to make is delivered succinctly and convincingly. 

Major comments
- It is tempting to use the argument of the paper as evidence for need of accurate ∆∆G predictions, i.e. ascertaining the impact of individual mutations on their core biochemical function, rather than end phenotype. Is it known what fraction of ∆∆G to phenotype relationships are non-monotonous? Of these, how well do ∆∆G changes need to be predicted in silico for a mutation to be able to correctly place it one this relationship curve? What is the combination of measurement accuracy and prediction ability that would allow making fundamental progress on the problem? Which data are most lacking (isolated structures, heterodimer structures, with/without ligand structures, etc)? What is the spectrum across biochemical events that we can predict well (a single binding event of two molecules in vitro), have some handle on (TF to DNA binding in a particular cell type), and have little hope to predict?

- It is challenging upon first reading to understand the exposition from the first Prm analysis. For example, L118, considering "mutations with measured effect on Prm expression". Does "measured effect" also mean knowing the ∆∆G to expression change function, as well as the ∆∆G of the mutation, or knowing the expression change from wild type only? 


Minor comments
- It took me a while to understand the context - one can measure expression changes, and has to infer coordinates in the biochemical space from it. Perhaps more natural in this case to swap the axes in some of the plots to drive the message home? Both multiple options for one difference in expression for Prm, and large changes in ∆∆G due to small measurement differences in expression would be have [for this reviewer] a more intuitive presentation (expression change acts as input in this setting, ∆∆G as output). This is a suggestion only.


Reviewer #3 (Remarks to the Author):

In this manuscript by Li and Lehner titled “Biochemical ambiguities prevent accurate genetic prediction”, the authors demonstrated that prediction of the combined phenotypic effect of two mutations from experimentally measured individual phenotypes of each of the mutations is often unattainable due to 1) one-to-many mapping of phenotype to biochemical properties of a single mutation, in particular when mutations are accompanied by biochemical pleiotropy; and 2) non-monotonicity of input-output function that relates a biochemical property (e.g., protein concentration) and a phenotype (e.g., gene expression). The authors based their conclusion on a well-established thermodynamic model that predicts the probability of expression from two promoters, PR and PRM, as a function of concentration and free energies of dimerization, DNA binding, and tetramerization of CI repressor. The authors modified the model by adding two additional thermodynamic states, folded and unfolded CI monomer.

Major points
The realization that a wide-range of mutation-induced perturbations of the biochemical parameters of a protein can all result in an identical phenotype is not new and was demonstrated already in 1985, when multiple mutations greatly affecting catalytic parameters (kcat, Km) of an enzyme were shown to have a similar effect on metabolic flux (Genetics 111: 655-74). It is therefore unsurprising that the difficulty to precisely predict the biochemical properties underlying a given phenotype of a single mutation will be further exacerbated when the prediction of a combined phenotypic effect of two mutations is attempted.

Abstract.
The abstract is written in a very vague fashion making it difficult to precisely grasp what are the major findings of the paper. It should be explicitly stated that the prediction of the combined phenotypic effects (instead using an ambiguous “outcome”) of two mutations with known phenotypes is thwarted by biochemical pleiotropy of mutations and the non-monotonicity of input-output function. Further, I am not sure that the phrase “the combined effects of genetic variants can be determined from their individual effects on a trait” is a widely accepted notion, as stated in lines 14-15. On the contrary, multiple publications, including the ones cited in this manuscript, point out that combination of known genetic traits relate to phenotypes in a very non-trivial way.

Terminology.
1. Strictly speaking, both the molecular properties of the CI repressor and gene expression from PR and PRM promoters are ‘phenotypes’. In the Discussion, phenotypes are divided into “higher-level phenotypes”, and, when referring to CI concentration, “intermediate molecular phenotype” (lines 294, 295). I suggest to provide a clear definition of what is considered a ‘phenotype’ (either measurable or predicted) already in the Introduction, and then adhere to this definition throughout the manuscript.
2. Since the molecular parameters considered are rooted in a thermodynamic system (free energies of folding, dimerization, binding, etc), it is more appropriate to refer to them as biophysical and not ‘biochemical’.

Model.
1. The extensively studied thermodynamic model by Ackers (ref.16) was extended by adding two thermodynamic states: unfolded and folded CI monomer. This extension is based on the assumption that monomer folding and dimerization are an uncoupled process, that is folding/unfolding is a unimolecular reaction that precedes the dimerization of two folded monomers (a bimolecular reaction). But is this assumption true? There are many examples, e.g., P22 Arc repressor (Biochemistry 1994, 33: 1125-1133), when folding and dimerization are coupled. If folding and dimerization are coupled, the proposed extension of the model does not capture correctly the concentration of Cl dimers as a function of total Cl molecules.
2. What is the reason for not including the unfolded and folded states in the partition function (i.e., the denominator) in Eq. 6 and 7?
3. Is there an experimental evidence that can corroborate that the extension of the Ackers’ model (i.e., the addition of folded and unfolded states) can indeed predict the expression state of PR and PRM promoters from a validated molecular effect of a mutation? Specifically, can the extended model correctly predict gene expression from ddG of folding for mutations whose effects on folding and on gene expression are known? Such an experimental corroboration is necessary to convince that the proposed extended model indeed captures the molecular mechanisms underlying the regulation of expression from PR and PRM promoters.
4. It is suggested that there might be mutations that affect only the free energy of folding/unfolding without changing the dimerization, tetramerization, or DNA binding of the repressor. Although it is indeed true that mutations can affect dG of folding/unfolding without changing other energy terms, change in the concentration of a folded monomer will affect (through mass action) the concentration of a dimer, and as a result will inevitably affect the dimerization, tetramerization, and DNA binding propensity. It is therefore not correct to treat the dG of folding/unfolding as a fully independent term.

Figures.
Fig. 2. E-H. What are the 4 different phenotypes of expression from a single promoter? The Acker’s model calculates the probability of gene expression, meaning that the phenotype is binary, either ON or OFF. What are the other two? Additionally, it is not entirely clear what is meant by the “expression range” between 0 to >20? (the probability of expression ranges from 0 to 1).

**Author rebuttal, first version:**

Reviewer #1 (Remarks to the Author):

Thank you for the opportunity to review “Biochemical ambiguities prevent accurate genetic prediction” by Li and Lehner. This manuscript reports on the challenges to predict phenotype resulting from a combination of mutations by only relying on their individual effects without additional phenotypic measurements. Authors use phage lambda repressor (CI), a well-studied protein and a model for gene regulation, to computationally model the expression of its two target genes using a thermodynamic model. They tested how mutations in CI that exert different biochemical effects, specifically its binding affinity or protein stability, affect target gene expression. They show that one-to-many mapping between phenotypes, made it impossible to predict how two mutations in CI combine without incorporating biochemical measurements that resolve ‘biochemical ambiguities’.

This study investigates how predictable the effect of combinations of mutations is on cellular phenotypes. This addresses an important question in the field of genetic interactions and provides novel insight about the difficulty to make such predictions based on sole phenotypes of single mutants without other measurements, in this case biochemical. Overall, this is a really nice paper.

We thank reviewer #1 for the enthusiasm and insightful and helpful comments. Please see below for our replies to the suggestions and comments.

Specific comments:

1. The introduction should describe Acker’s statistical thermodynamic model since the manuscript is rooted in this model.

We thank the reviewer for this suggestion and have included a brief description of Ackers’ statistical thermodynamic model in the introduction. A more detailed description is still provided in the ‘methods’ section.

We have added the following text to the introduction (Line 89):

“Specifically, Ackers’ statistical thermodynamic model predicts the probabilities of the ‘on’ and ‘off’ configuration states of the PR and _PRM_ promoters as a function of the total repressor concentration (Fig. 1B)^17^. To predict how mutations that affect the stability of CI combine to affect gene regulation, Ackers’ model can be combined with a thermodynamic model of protein folding^19^. ”

2. In Fig. 1D, it is not clear what the dotted vertical black line is supposed to represent.

We added ‘Dotted vertical black lines denote ∆∆G=0 (wild type)’ to the figure legend.

3. In Fig. 4A-B single mutation is denoted as ‘x’ in the figure legend however the figure itself uses ‘+’. Authors should be consistent with their notation.

We changed ‘x’ to ‘+’ in the legend.

4. In Fig. 4C-J, I had a difficult time differentiating the grey ‘x’ from everything else in the plot.

We remade the plot so that ‘x’ is in the white color to be easily distinguishable from the rest.

5. Authors should incorporate into the discussion, what would be the expected behaviour of heteromers and multisubunit protein complexes given their findings about CI homomers.

We thank the reviewer for this suggestion and have added the following text to the discussion (Line 346):

“The extent to which biophysical ambiguities will thwart the prediction of different phenotypes will depend on the number of parameters that can be affected by mutations, the biophysical pleiotropy of mutations, and the monotonicity of input-output functions. The distributions of mutational effects on multiple biophysical parameters have been quantified for very few systems, but for both the lambda repressor and other proteins, mutations frequently affect both stability ^41,42^ and binding to interaction partners ^41,43,44^ with biophysical pleiotropy and non-monotonic functions also common ^31,35,45.^ In other words, we expect biophysical ambiguity to confound phenotypic prediction in other systems including heteromeric complexes and beyond transcription factor-mediated repression.”

Reviewer #2 (Remarks to the Author):

In this paper, the Authors demonstrate that it can be impossible to predict the joint effect of two mutations in one genetic background from their individual effects observed in isolation. They use the classical lambda repressor system that is very well understood, to make this point explicitly.

To some extent, the claim is trivial - mathematically, not all functions are injective; statistically, marginal distributions of two variables are not generally sufficient to reconstruct their full joint distribution; in genetics, interactions have been appreciated for over a century. The novelty is demonstrating that knowing the mechanism does not rescue this ability, even in the simplest known two-stage systems.

In a sense, the manuscript raises more questions than it answers, but perhaps this is not a bad thing. The point it does attempt to make is delivered succinctly and convincingly.

We thank reviewer #2 for the insightful and thoughtful comments. Please see below for our replies to the suggestions and comments.

Major comments

- It is tempting to use the argument of the paper as evidence for need of accurate ∆∆G predictions, i.e. ascertaining the impact of individual mutations on their core biochemical function, rather than end phenotype. Is it known what fraction of ∆∆G to phenotype relationships are non-monotonous? Of these, how well do ∆∆G changes need to be predicted in silico for a mutation to be able to correctly place it one this relationship curve? What is the combination of measurement accuracy and prediction ability that would allow making fundamental progress on the problem? Which data are most lacking (isolated structures, heterodimer structures,

with/without ligand structures, etc)? What is the spectrum across biochemical events that we can predict well (a single binding event of two molecules in vitro), have some handle on (TF to DNA binding in a particular cell type), and have little hope to predict?

We agree with the referee. To resolve the prediction ambiguity in general, what we need most are high throughput measurements of the mutational effects on different biophysical properties, not just cellular or organismal phenotypes. For example, to distinguish the effects of mutations on binding affinity and protein stability we need to measure their effects on both binding to an interaction partner (for example the concentration of a complex) and the change in the concentration of the free monomer. From these two measurements, we can infer the free energy changes of protein stability and binding by fitting a global model. We are actively developing generic methods to address this challenge and have highlighted the challenge in the Discussion.

As also now mentioned in the Discussion, non-monotonic dose-response relationships are commonly observed in biology. For instance, a systematic study on relationships between protein expression levels and yeast fitness shows that about 35% of the 81 examined dose-response curves are non-monotonic (Karen [et.al](http://et.al),

2016, Cell 166). Drug/inducer dose - response relationships are also frequently non-monotonic (Varret et al., 2018, Toxicology and applied pharmacology 339, 15). We thus expect non-monotonic ∆∆G-phenotype functions to be quite widespread. However, whether detrimental mutations (increases in ∆∆G) combine according to a non-monotonic relationship depends upon where the wild-type protein sits on the function.

- It is challenging upon first reading to understand the exposition from the first Prm analysis. For example, L118, considering "mutations with measured effect on Prm expression". Does "measured effect" also mean knowing the ∆∆G to expression change function, as well as the ∆∆G of thmutation, or knowing the expression change from wild type only?

Here we meant knowing the expression change from the wild type. We have re­written this sentence as follows:

“Combining two mutations with measured effects on _PRM_ expression can result in more than one _PRM_ expression value, depending upon what the hidden underlying free energy changes are 19,40. ”

Minor comments

- It took me a while to understand the context - one can measure expression changes, and has to infer coordinates in the biochemical space from it. Perhaps more natural in this case to swap the axes in some of the plots to drive the message home? Both multiple options for one difference in expression for Prm, and large changes in ∆∆G due to small measurement differences in expression would be have [for this reviewer] a more intuitive presentation (expression change acts as input in this setting, ∆∆G as output). This is a suggestion only.

We thank the reviewer for this suggestion. We plotted ∆∆G values as the x-axis values and phenotypes as y-axis values in the plots because we thought researchers in the genotype-phenotype mapping and deep mutational scanning fields would be more used to this orientation. However, we agree with the reviewer that swapping the axes might also help clarify the analyses. Hence, we modified Supplementary Figure 1, including figure panels with the swapped axes (panels B- E). In this revised figure, we show mapping phenotypes (on the x-axis) from a single mutant to ∆∆G (on the y-axis), and how ∆∆G values are used to calculate double mutants’ phenotypes.

Reviewer #3 (Remarks to the Author):

In this manuscript by Li and Lehner titled “Biochemical ambiguities prevent accurate genetic prediction”, the authors demonstrated that prediction of the combined phenotypic effect of two mutations from experimentally measured individual phenotypes of each of the mutations is often unattainable due to 1) one-to-many mapping of phenotype to biochemical properties of a single mutation, in particular when mutations are accompanied by biochemical pleiotropy; and 2) non-monotonicity of input-output function that relates a biochemical property (e.g., protein concentration) and a phenotype (e.g., gene expression). The authors based their conclusion on a well-established thermodynamic model that predicts the probability of expression from two promoters, PR and PRM, as a function of concentration and free energies of dimerization, DNA binding, and tetramerization of CI repressor. The authors modified the model by adding two additional thermodynamic states, folded and unfolded CI monomer.

We thank reviewer #3 for the accurate summary of our work and insightful comments and suggestions. Please see below for our replies to the suggestions and comments.

Major points

The realization that a wide-range of mutation-induced perturbations of the biochemical parameters of a protein can all result in an identical phenotype is not new and was demonstrated already in 1985, when multiple mutations greatly affecting catalytic parameters (kcat, Km) of an enzyme were shown to have a similar effect on metabolic flux (Genetics 111: 655-74). It is therefore unsurprising that the difficulty to precisely predict the biochemical properties underlying a given phenotype of a single mutation will be further exacerbated when the prediction of a combined phenotypic effect of two mutations is attempted.

We thank the reviewer for the thoughtful comment. Indeed it has long been appreciated by at least some more thoughtful researchers that different combinations of biochemical parameters can result in the same or similar phenotypic values. We have added a citation to the Hartl paper to the third paragraph of the introduction.

In a way, what we are demonstrating here is the implications of this for the analysis and prediction of genetic interactions (epistasis): ynless we know (or can infer) which parameter(s) two mutations have affected we often cannot predict what happens to a phenotypic trait when they combine, even if we have very precise phenotypic measurements and we know how different biophysical parameters influence the phenotype. This, unfortunately, is widely overlooked by geneticists.

“In this study, we use a simple biophysical system to address this question. We show that, for diverse biological systems, the answer to this question will often be ‘no’. The fundamental reason for this is that different combinations of biophysical parameters can give rise to the same phenotypic value ^9^.”

Abstract.

The abstract is written in a very vague fashion making it difficult to precisely grasp what are the major findings of the paper. It should be explicitly stated that the prediction of the combined phenotypic effects (instead using an ambiguous “outcome”) of two mutations with known phenotypes is thwarted by biochemical pleiotropy of mutations and the non-monotonicity of input-output function. Further, I am not sure that the phrase “the combined effects of genetic variants can be determined from their individual effects on a trait” is a widely accepted notion, as stated in lines 14-15. On the contrary, multiple publications, including the ones cited in this manuscript, point out that combination of known genetic traits relate to phenotypes in a very non-trivial way.

We thank you for the suggestion and have edited the abstractL

“A central goal of genetics is to predict how mutations combine to alter phenotypes, fitness and disease. It is widely assumed in quantitative genetics that mutations combine additively or that the genetic interactions between them (epistasis) can be learnt and predicted using statistical or mechanistic models. Here, we show that, even for the simple system of a transcription factor repressing a target gene, this assumption is incorrect and that perfect measurements of the effects of mutations on a trait and a mechanistic understanding of a system can be insufficient to predict what happens when two mutations are combined. This apparent paradox arises because mutations can have many different biophysical effects to cause the same change in a phenotype. When combining mutations, the joint effect on a trait can be very different depending upon what these hidden biophysical changes actually are. Biophysical pleiotropy and non-monotonic functions further confound prediction of how mutations interact. We suggest that accurate genetic prediction of phenotypes and disease will sometimes not be possible unless these biophysical ambiguities can be resolved by additional measurements.”

Terminology.

1. Strictly speaking, both the molecular properties of the CI repressor and gene expression from PR and PRM promoters are ‘phenotypes’. In the Discussion, phenotypes are divided into “higher-level phenotypes”, and, when referring to CI concentration, “intermediate molecular phenotype” (lines 294, 295). I suggest to provide a clear definition of what is considered a ‘phenotype’ (either measurable or predicted) already in the Introduction, and then adhere to this definition throughout the manuscript.

Following the reviewer’s suggestion, we have clarified this when the PR and PRM phenotypes are first mentioned (Line 141). :

“Unless they are energetically coupled ^37^, mutations have effects on stability that are additive at the level of free energy but non-additive for changes in protein concentration and expression from the PR and _PRM_ promoters, which are our two phenotypic traits of interest (Fig. 1C, D) 19,38,39. ”

2. Since the molecular parameters considered are rooted in a thermodynamic system (free energies of folding, dimerization, binding, etc), it is more appropriate to refer to them as biophysical and not ‘biochemical’.

We agree and have changed ‘biochemical’ to ‘biophysical’ throughout the text and the figures, and also in the title.

Model.

1. The extensively studied thermodynamic model by Ackers (ref.16) was extended by adding two thermodynamic states: unfolded and folded CI monomer. This extension is based on the assumption that monomer folding and dimerization are an uncoupled process, that is folding/unfolding is a unimolecular reaction that precedes the dimerization of two folded monomers (a bimolecular reaction). But is this assumption true? There are many examples, e.g., P22 Arc repressor (Biochemistry 1994, 33: 1125-1133), when folding and dimerization are coupled. If folding and dimerization are coupled, the proposed extension of the model does not capture correctly the concentration of Cl dimers as a function of total Cl molecules.

We thank the reviewer for raising an important aspect of the model.

We agree that it is more likely that folding and dimerization are coupled into one equilibrium, and our model indeed treats CI protein folding and dimerisation as part of the same equilibrium i.e. as coupled reactions. We have clarified this in the Methods section (Line 341) and it is also shown in Figure 1B..

“We consider the system as a single equilibrium i.e. protein folding and dimerization are coupled reactions.”

We have also explored a model that treats folding and dimerization as separate equilibria, and compared the results to our current model. The conclusions are very similar because, as shown below, the free energy-phenotype functions are very similar in the coupled and uncoupled systems.

2. What is the reason for not including the unfolded and folded states in the partition function (i.e., the denominator) in Eq. 6 and 7?

The logic of not including the unfolded protein state in Eq. (6) and (7) in our model is the same as for not including the monomer protein state in the corresponding equation in Ackers’ original model (Below, Eq. [1] from Ackers et al., 1982, PNAS 79, ‘fs’ is the probability of the operator in the ‘s’ configuration. ‘[R2]’ is the free dimer concentration. This equation for the *promoter* states does not include the CI monomer state; the CI dimer concentration is used as the reference state.

Eq. (6) and (7) from our methods section (shown below) are the probabilities of PR promoter and _PRM_ promoter to be ON. These equations follow Ackers’ original model.

3. Is there an experimental evidence that can corroborate that the extension of the Ackers’ model (i.e., the addition of folded and unfolded states) can indeed predict the expression state of PR and PRM promoters from a validated molecular effect of a mutation? Specifically, can the extended model correctly predict gene expression from ddG of folding for mutations whose effects on folding and on gene expression are known? Such an experimental corroboration is necessary to convince that the proposed extended model indeed captures the molecular mechanisms underlying the regulation of expression from PR and PRM promoters.

A brief answer to the reviewer's question is yes.

In our previously published study (Li et al., 2019, Nature Communications 10.1038/s41467-019-11735-3), we constructed more than 800 single and double amino acid substitutions in the CI helix-turn-helix DNA-binding domain, and used GFP expression under the control of the PR promoter as a reporter for the phenotype (PR expression). We performed the experiments at two concentrations of CI.

In order to predict how mutations combine based on the individual mutants’

phenotypes, we found that extending Ackers’ thermodynamic model to include the folded and unfolded states of CI was necessary. Using Ackers’ original model, we could explain ~40% and <5% of total explainable variance in double mutant phenotypes at low and high concentrations of CI respectively. With a full model combining the thermodynamics of protein folding with Ackers’ model we were able to explain 78% and 90% of the total explainable variance in the double mutants’ phenotypes (Figure 3f of the earlier paper). This improvement in explaining the data was due to better prediction of the underlying additive biophysical trait, which was not accurately predicted with Ackers’ model alone (Supplementary Figure 5 of the paper).

The assumption of our model in that study was that the majority of mutations in this domain primarily affect protein folding, and there is generally good agreement with this. However, there is still plenty of unexplained variance in the data, consistent with the literature showing that mutations affect additional biophysical parameters and sometimes more than one biophysical parameter.

4. It is suggested that there might be mutations that affect only the free energy of folding/unfolding without changing the dimerization, tetramerization, or DNA binding of the repressor. Although it is indeed true that mutations can affect dG of folding/unfolding without changing other energy terms, change in the concentration of a folded monomer will affect (through mass action) the concentration of a dimer, and as a result will inevitably affect the dimerization, tetramerization, and DNA binding propensity. It is therefore not correct to treat the dG of folding/unfolding as a fully independent term.

As the reviewer pointed out, and also as we stated in response to comment 1, concentrations of CI in different states are not independent. In other words, changing the folding energy will affect not only the folded monomer concentration, but also the dimer concentration. Our model took this into account, by coupling all the reactions in a single equilibrium.

In the revised manuscript, we have explicitly stated this in a sentence in the Methods section (Line 342):

“We consider the system as a single equilibrium i.e. protein folding and dimerization are coupled reactions.”

Figures.

Fig. 2. E-H. What are the 4 different phenotypes of expression from a single promoter? The Acker’s model calculates the probability of gene expression, meaning that the phenotype is binary, either ON or OFF. What are the other two? Additionally, it is not entirely clear what is meant by the “expression range” between 0 to >20? (the probability of expression ranges from 0 to 1).

The reviewer is correct - Ackers’ model calculates the probability of gene expression, which ranges from 0 to 1. The phenotype we use is also this probability rather than a binary ON (1) or OFF (0) state. The number of different phenotypes from a single promoter in Fig. 2 E- H indicates the number of distinct phenotypic values (the probability of the promoter being ON).

Fig. 2I - K are shown only to explain Fig. 2E- H by giving specific examples. For example, the last column of Fig. 2I, corresponding to the segment indicated by the right-most grey dashed vertical lines in Fig. 2E, shows how a single mutation A with the PRM expression of -1 (in log(2) scale) combines other single mutations of a given phenotype. As you can see in this particular case, there are always at least two different double mutant phenotypes (y-axis) for a given single mutant phenotype (x-axis). When the single mutant’s PRM expression is above -2.5 (in log(2) scale), there are 4 possible double mutant phenotypes (4 different y-axis values for a common x-axis value).

Similarly, ‘expression range’ means the differences between maximum and minimum gene expression levels from the same-phenotype combinations. As we considered phenotypes in the log(2) scale, the maximum gene expression level becomes 0, but

**the minimum gene expression is below 0 without a lower limit. Therefore, gene expression range can be > 20 (for example, if the minimum gene expression is < -20 while the maximum gene expression = 0). We modified Fig. 2 to make this point clearer.**

**Reviewer comments, second version:**

Reviewer #1 (Remarks to the Author):

The authors have sufficiently addressed most comments.


Reviewer #2 (Remarks to the Author):

The Authors have addressed all my comments.


Reviewer #3 (Remarks to the Author):

The authors have addressed my questions and suggestions, I do not have any further comments

**Author rebuttal, second version:**

**Reviewer comments, third version:**

**Author rebuttal, third version:**
